# Hydrophilization of Polypropylene by Gaseous Plasma Treatments and Hydrophobic Recovery

**DOI:** 10.3390/polym17192644

**Published:** 2025-09-30

**Authors:** Gregor Primc

**Affiliations:** Department of Surface Engineering, Jožef Stefan Institute, Jamova Cesta 39, 1000 Ljubljana, Slovenia; gregor.primc@ijs.si

**Keywords:** polypropylene, gaseous plasma, hydrophilization, hydrophobic recovery

## Abstract

Although polypropylene (PP) is among the most widely used polymers with adequate chemical and mechanical properties, its poor wettability prevents adhesive joints needed for sticking with other materials, printing, etc. Plasma treatment, an established method for increasing wettability, is presented, and relevant literature is analyzed. A comparison of different reviewed articles shows little influence of the discharge parameters on PP wettability, and that the methods for achieving a super-hydrophilic surface of this polymer have yet to be developed. The peculiarities of PP prevent stable surface functionalization, although the formation of molecular fragments is the predominant effect of plasma treatments. The key conclusion after analyzing the reviewed literature is that the washing of PP following plasma treatment will cause a low level of wettability regardless of the peculiarities of the plasmas or discharges, including the treatment time, and all authors reported a water contact angle between about 75 and 80° after washing the plasma-treated PP. The hydrophobic recovery of washed plasma-treated PP was not addressed in any reviewed article.

## 1. Introduction

Polypropylene (PP) is a commonly used polymer with broad applications from electronics to the food industry and medical products [1,2,3]. Its wide application arises from its low cost, good chemical inertness, and ease of manufacturing various products, from textiles to three-dimensional objects. The chemical inertness may be an obstacle in numerous applications, such as when a polypropylene product is printed, painted, or joined to another object by gluing. Good chemical inertness causes poor interaction of the coating on the PP product, and the absence of adhesion forces is attributed to the lack of polar functional groups on the PP surface. The adhesion of the coating could be increased by treating the PP surface with polar functional groups. Several methods for the surface functionalization of PP products have been reported [4], including the thermo-oxidative chemical method [5], thermo-molecular adhesion [6], flaming, side-chain crystalline block copolymer [7], and photochemical metallization [8]. An often used method is treatment with gaseous plasma. Namely, plasma treatment does not require any chemicals and produces no waste, and thus is regarded as ecologically benign. Both atmospheric and low-pressure plasmas are useful for modifying polymer surface properties. Atmospheric pressure plasmas are also cost-effective because the discharges are powered by inexpensive generators and vacuum pumps are not needed, albeit one drawback may be the inability to sustain homogeneous non-equilibrium plasma in a large volume. Accordingly, such plasmas are not very useful for treating three-dimensional products [9]. Even though this limitation does not apply to low-pressure plasmas, several technological challenges still arise when seeking to apply such plasmas on an industrial scale [10].

Plasma treatment of PP was first reported decades ago. Early scientific articles include [11,12,13], and perhaps the first theory on surface kinetics on the atomic scale was provided by a team led by Kushner [14]. Later, several authors reported plasma functionalization using various experimental setups. Knowledge concerning the surface kinetics upon plasma treatment of PP has expanded over the decades. Today, it is generally accepted that plasma treatment causes numerous effects, including surface functionalization with polar functional groups. Other reactions include chain scission, cross-linking, modification of a surface film’s mechanical properties, formation of low-molecular-weight fragments, formation of volatile molecules with high vapor pressure even at room temperature, and etching, which typically modifies the surface morphology [6,15,16,17,18,19,20,21]. All these reactions influence the performance of plasma-treated PP. The intensity of various reactions occurring on the PP surface or subsurface region depends on the plasma parameters, which, in turn, depend on how the experimental setup was configured by the authors. Unfortunately, the vast majority of authors who reported the surface functionalization of PP by gaseous plasma treatment did not even mention the plasma parameters, let alone the intensity of a particular surface reaction versus the fluxes and/or fluences of the plasma species. The plasma species capable of causing the surface modification of PP include positively charged ions and neutral radicals, such as atoms, highly excited molecules, and energetic photons. The lack of detailed knowledge on the interaction of plasma species with the PP surface is not simply due to inadequate plasma characterization but also the lack of techniques for real-time monitoring of the surface composition and structure during plasma treatment. Namely, the vast majority of authors did not integrate the plasma into instruments for surface and thin film characterization. Instead, they treated polymer samples in a plasma reactor, exposed them to air, and performed surface characterization several minutes or even hours after the plasma treatment. Any fast surface reactions will occur between the plasma treatment and the surface characterization, and thus some information on the surface kinetics will be lost before the plasma-treated material is characterized. Spontaneous reactions cause a relaxation of the plasma-induced surface effects and, thus, aging. Aging is generally attributed to hydrophobic recovery, that is, the spontaneous loss of the hydrophilic character of the plasma-treated PP.

Aging is particularly apparent in systems with high potential energy or large gradients in the concentrations of various elements and functional groups. The laws of thermodynamics facilitate spontaneous relaxation until the most stable form has been reached. Gaseous plasma is a source of particles holding large potential and/or kinetic energy, and the vast majority of their interaction with the solid material occurs right on the surface such that the plasma treatment causes gradients and, thus, a thermodynamically unfavorable surface finish. Despite the aging of polypropylene being unavoidable, it is manageable to a tolerable extent, as shown in this review article.

The vast majority of authors used one of the oldest and most reliable methods for detecting the aging phenomenon of plasma-treated polymers: a water droplet. Only a few authors relied on more sophisticated methods, and some results are contradictory. It is hence impossible to draw any correlation between these methods and the water-droplet technique.

## 2. Gaseous Plasma

Gaseous plasma is the most common state of matter in the universe. In thermally equilibrium plasma, the distribution of excited states of gaseous molecules follows the Boltzmann and Maxwell laws, which means the concentration of plasma species with high potential or kinetic energy is only achievable at high gas temperatures. Polymers degrade at high temperatures and thermal plasma has limited use as it can only tailor the surface properties of PP when applied in very short pulses.

Non-equilibrium plasma is more useful for tailoring the surface properties of polymers. It is usually sustained by a gaseous discharge. A sufficiently high electric field is applied, and the charged particles accelerate and gain energy from that field. Molecular ions quickly lose their kinetic energy in elastic collisions with neutral molecules, radicals, and atoms, while electrons maintain their energy and their temperature (or, better, distribution over the kinetic energy) remains much larger than the temperature of other particles. In fact, the electron temperature in most plasmas of use for treating polymers is several 10,000 K. Such a high electron temperature does not cause significant heating of polymer samples exposed to non-equilibrium gaseous plasma for two reasons: first, the electron density is typically much smaller than the density of gaseous molecules and radicals, and second, they are retarded from the surface due to the negative surface potential, which is spontaneously formed upon the treatment of any object with non-equilibrium plasma. The role of free electrons in plasma is the excitation of molecules, which may be excited to electronic and rovibrational states upon inelastic collisions with such electrons. Energetically sufficient electrons may cause the dissociation of gaseous molecules, and electrons from the high-energy tail of their distribution function will also cause ionization. Ionization is crucial for sustaining gaseous plasma since the production of electrons upon ionization collisions between fast electrons and neutral particles should compensate for the loss of electrons by neutralization with positively charged ions.

The density of neutral plasma species with high potential energy (radicals, electronically excited molecules in metastable states, and atoms) is generally orders of magnitude larger than the electron density because the loss in the gas phase or on surfaces is often marginal compared to the neutralization of the charged particles. Electrons from the high-energy tail of their energy distribution also excite energetic states that are unstable, and relax almost immediately by electric dipole radiation. Therefore, non-equilibrium gaseous plasma is also a rich source of radiation, which is often predominant in the vacuum ultraviolet (VUV) range of the spectrum; as in, below approximately 200 nm. Such photons are energetic enough to cause bond scission in polymer materials. The concentration of electrons capable of exciting radiative energetic states is low and therefore the flux of VUV photons is much smaller than that of the reactive plasma particles suitable for chemical interaction with the PP samples. However, it is not negligible and causes significant modification of the polymer surfaces and subsurface film of thickness, which corresponds to their penetration depth.

Non-equilibrium plasma is usually sustained by an electrical discharge, in either a reactor in a controlled atmosphere or ambient conditions. The adjustable (also sometimes known as ‘external’) parameters include the peculiarities of the plasma reactor and the discharge configuration, discharge voltage, current, power, types of gases and their flow rates, and gas pressure. The discharge causes the formation of plasma, which is characterized by plasma parameters such as the electron density and temperature (or electron energy distribution function), density of positively and negatively charged molecules and their radicals, and density of metastable atoms and molecules. Plasma species impinge on the polymer surface, and thus the polymer is subjected to a flux of such species.

**Figure 1:**
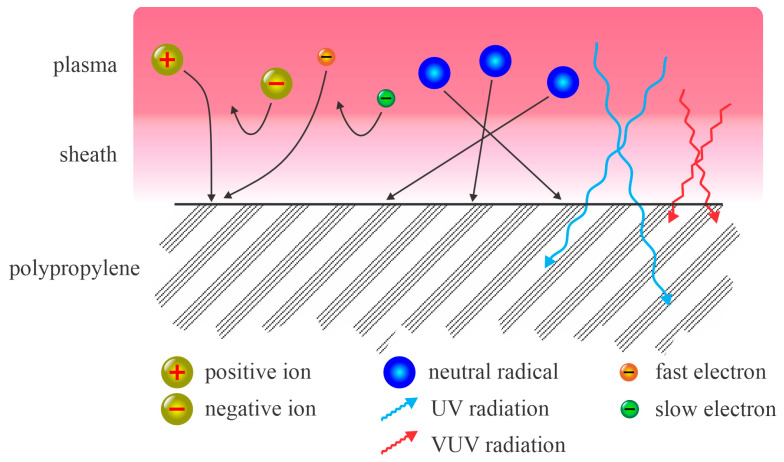
Illustration of the plasma treatment of PP in a collisionless sheath approximation. Black arrows indicate the path of plasma particles (electrons, ions, and neutral radicals).

The treatment of PP with non-equilibrium plasma is illustrated in Figure 1. A sheath exists between the plasma and the polymer surface. The electrons are much faster in the plasma than any other charged particles. Accordingly, the first effect after turning on the discharge is the formation of a negative charge on the polymer surface facing the plasma. As a result, the electric field retards the negatively charged plasma species and accelerates the positive ions. The positively charged ions are thus accelerated across the sheath and bombard the polymer’s surface. Their kinetic energy, however, is low, typically about 10 eV less when they collide with slow neutrals while passing the sheath. The electrons are retarded by the potential across the sheath and therefore only those from the high-energy tail can reach the surface. This explains why their influence on polymer modification is typically minor. The same applies to negatively charged ions, which are present in significant concentrations in plasmas sustained in electronegative gases. Since their kinetic energy in the plasma is low, much lower than the electron energy, they are unlikely to reach the polymer surface. The neutral species usually move randomly in both the plasma and the sheath; hence, they impinge on the surface in all directions. Their temperature in non-equilibrium plasma is low and remains low within the sheath, which means they do not have substantial kinetic energy while impinging on the polymer surface. Metastables may release their potential energy upon colliding with a polymer surface. Finally, radiation arising from the plasma influences the polymer if the photon energy is above the threshold for bond scission. The penetration depth of radiation exceeds that of the other plasma species. Figure 1 indicates that the fluxes of positively charged ions, neutral radicals, and UV/VUV radiation govern the polymer surface finish.

**Figure 2:**
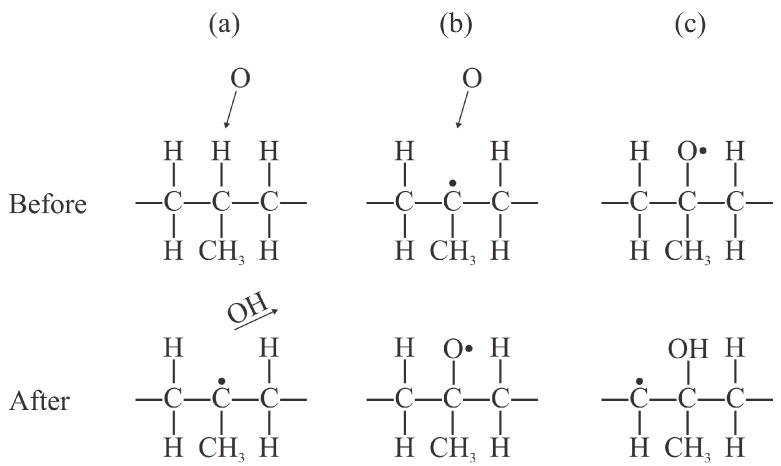
The initial interactions on the PP surface upon exposure to O atoms: (**a**) formation of a dangling bond and desorption of an OH radical; (**b**) formation of an alkoxy radical; and (**c**) formation of a hydroxyl functional group.

Numerous surface reactions occur when treating polypropylene with plasma species. As early as in 2003, Dorai et al. identified about 20 different surface reactions caused by oxygen species [14]. The initial reactions while treating polypropylene with oxygen plasma are the subtraction of an H atom and the formation of an OH radical, which desorbs from the surface. The resulting dangling bond is likely to be occupied by another oxygen atom from the gas phase. According to Dorai et al., the dangling bond is sufficiently reactive to interact with ozone and even neutral oxygen molecules in the ground electronic state [14]. The initial reactions likely to occur upon interaction of PP with oxygen plasma are shown in Figure 2. Once the surface is saturated with hydroxyl groups, other groups are likely to be formed upon further exposure to O atoms, and the bond scission in the polymer backbone is likely to occur in the surface film with a high oxygen concentration, which leads to etching (the release of CO_2_ and H_2_O molecules).

## 3. Methods for Surface Characterization of the Aging Effects of Plasma-Treated Polypropylene

**Figure 3:**
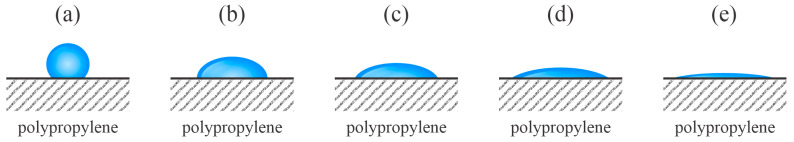
Photographs of water droplets on PP surfaces with different wettability: (**a**) untreated; (**b**) weakly hydrophilized; (**c**) moderately hydrophilized; (**d**) highly hydrophilized; and (**e**) super-hydrophilic.

The choice of methods suitable for studying the aging of plasma-treated PP is limited. The simplest is the deposition of a water droplet and measurement of the contact angle of the droplet. This method is simple for interpretation provided that the sample is perfectly smooth and the surface composition and structure are homogeneous. The interpretation of the droplet contact angle may be very complicated if these conditions are not satisfied, particularly for polymer materials with complex morphologies [22]. A large water contact angle (WCA) indicates the hydrophobic character of the substrate, and a material with better wettability exhibits a WCA well below 90°. If the water contact angle is immeasurably low, namely, if the droplet spreads over a large surface, it is impossible to measure its contact angle, and the material is super-hydrophilic. Although the superhydrophilicity criterion is somewhat arbitrary, multiple authors have used this expression for materials exhibiting a WCA below 7°. Such a small WCA can be measured using professional drop-shape analyzers. Photographs of water droplets on PP surfaces with different degrees of plasma-induced wettability are shown in Figure 3.

Other methods for evaluating the hydrophilicity of plasma-treated PP do not detect or measure wettability but consider property that dictates hydrophilicity. These include methods for measuring the surface composition, structure, and morphology. The most widely used techniques include X-ray Photoelectron Spectroscopy (XPS), Secondary Ion Mass Spectrometry (SIMS), Fourier-transform Infrared (FTIR) absorption, Atomic Force Microscopy (AFM), and Scanning Electron Microscopy (SEM). These techniques complement WCA and are often used to probe the surface hydrophilicity of various materials. A recent review of methods for determining polymer aging is provided by Tian et al. [23].

## 4. Review of Articles Reporting the Hydrophobic Recovery of Plasma-Treated PP

**Figure 4:**
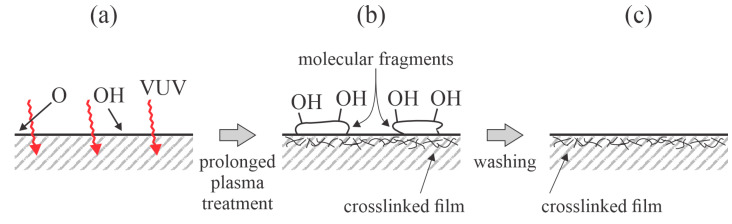
Illustration of surface effects reported by the pioneer team on PP hydrophobic recovery: (**a**) PP is exposed to a random flux of VUV radiation and radicals (largely O atoms in the case of oxygen plasma and OH radicals in the case of hydrogen plasma); (**b**) prolonged treatment causes bond scission by absorption of VUV radiation, cross-linkage, and the formation of molecular fragments that are functionalized with polar groups; (**c**) washing removes the functionalized molecular fragments such that the wettability is practically the same as that of the untreated PP samples. Only the crosslinked film persisted after washing.

One of the first studies on the aging of plasma-treated PP samples treated with gaseous plasma was conducted by Behnisch et al. [24]. The PP samples were first cleaned in an ethanol ultrasound bath and then exposed to non-equilibrium plasma treatments. A set of experiments was performed using solely oxygen plasma. Another set of samples was first treated with hydrogen plasma, and then with oxygen plasma. Hydrogen plasma is a rich source of VUV radiation [25]. The plasma reactor was evacuated using a rotary pump, and the ultimate pressure was 0.1 Pa. The plasma was sustained by a capacitively coupled radiofrequency (RF) electrodeless discharge, and the plasma reactor was made of a dielectric material (Pyrex glass). The density of the charged particles was negligible with the position of the samples in the plasma reactor as they were mounted far from the glowing plasma in the volume between the externally mounted electrodes. Indeed, the main plasma species that influenced the PP wettability in [24] were neutral radicals (H or O atoms) and quite weak radiation, which arose from the glowing plasma. The treatment time was 10 min for hydrogen plasma and 5 min for oxygen plasma. Interestingly, both treatments in oxygen plasma only and the subsequent treatments in hydrogen and oxygen plasma caused the same wettability since the WCA decreased from the initial 90° to approximately 50° immediately after the plasma treatments. The authors explained the moderately low WCA by bond scission rather than surface functionalization. Bond scission is a consequence of the absorption of VUV radiation. The plasma-treated samples were washed in distilled water in an ultrasonic bath, which increased the WCA to approximately 80° for both sets of samples. The authors explained the hydrophobic recovery upon ultrasound treatment by removing weakly bonded low-molecular-density fragments that formed upon the plasma treatment. Later, numerous authors treated PP samples, with all reporting surface functionalization with polar oxygen-containing functional groups after treatment with oxygen plasma [26]. Even though hydrogen plasma should not cause oxidation of the polymer samples, the comparable WCA obtained by pre-treatment in hydrogen plasma can be explained by the presence of a residual atmosphere in the treatment chamber, which is water vapor in the case of hermetically tight vacuum systems. Water molecules dissociate under plasma conditions, and OH radicals readily interact chemically with the polyolefin surfaces, forming surface hydroxyl (and other) groups [27]. Oxygen plasma is a much weaker source of VUV radiation than hydrogen and thus bond scission by energetic photons should be less intense in the case of oxygen plasma treatment [28]. Given these facts, the results reported by Behnisch et al. [24] can be interpreted as displayed in Figure 4. The effect of VUV irradiation on surface hydrophilization and hydrophobic recovery was later elaborated by Truica-Marasescu et al. [29]. They used a VUV lamp to treat PP foils.

Novak et al. [30] used PP foils of different crystallinity, biaxially oriented and extruded, to study the long-term hydrophobic recovery after plasma treatment. The samples were treated in plasma sustained at atmospheric pressure by corona discharge in air. Regrettably, details of the discharge configuration, power, and treatment time were not disclosed. The samples were not washed after plasma treatment. The WCA after plasma treatment was approximately 68° and 50° for biaxially oriented and extruded PP, respectively. Aging was pronounced in the first month of being stored in ambient conditions, and after 1 year, the final WCA stabilized at 75° and 80°. The polar component of the surface free energy (PCSFE) was measured during the prolonged aging. The plasma treatment enabled PCSFE values of 9 and 14 mJ/m^2^ for the biaxially oriented and extruded samples, respectively. Aging was much more pronounced for the extruded samples since the PCSFE stabilized at approximately 6 and 3 mJ/m^2^ after several months of being stored under ambient conditions for biaxially oriented and extruded samples, respectively. This is one of the few articles to report variation in hydrophobic recovery for the same material, but with different crystallinities. The adhesion force of the polyvinyl acetate rose linearly as the polar component of the surface free energy increased, indicating the beneficial effect of plasma treatment on the adhesion of the two polymers. Similar results were published in [31].

Guimond and Wertheimer also used corona discharge in the air to sustain plasma and treat biaxially oriented PP foils [32]. The energy dissipated on the polymer surface varied by up to approximately 400 mJ/cm^2^. The WCA of the untreated samples was about 85°, rising with increasing discharge energy, and stabilized at approximately 65° after an energy dose of ~30 mJ/cm^2^. The concentration of oxygen in the surface film, as probed by XPS (several nanometers), was approximately 5, 9, and 11 at.% at the energy doses of 10, 100, and 430 mJ/cm^2^, respectively. Such nonlinear behavior is typical for plasma-treated polymers because of the surface saturation with polar functional groups at moderate doses of plasma radicals and etching and nanostructuring at larger doses. The authors [32] washed PP foils in deionized water after plasma treatment. The wettability of the washed samples was worse than that of the unwashed samples. The concentration of oxygen on the washed samples was almost equal (approximately 4 at.%) for all samples, irrespective of the energy dissipated on the surface upon plasma treatment. The authors explained this effect by the removal of weakly bonded low-molecular-density fragments that formed upon the plasma treatment, similar to Behnisch et al. [24]. Interestingly, the loss of hydrophilicity after washing was practically the same as that reported by Behnisch et al. [24]. The WCA of the washed PP samples was about 77°, irrespective of the energy dose upon plasma treatment. The effects of washing, as shown in Figure 4c, also apply to [32], albeit the discharge parameters were completely different in [32] than in [24]. Hydrophobic recovery was studied for unwashed samples kept in separate Petri dishes at atmospheric pressure, 23 °C, and 50% relative humidity. The loss of wettability was almost independent of the energy dose between 200 and 600 mJ/cm^2^. The WCA increased to 75° within 1 day and to 78° within 3 months.

**Figure 5:**
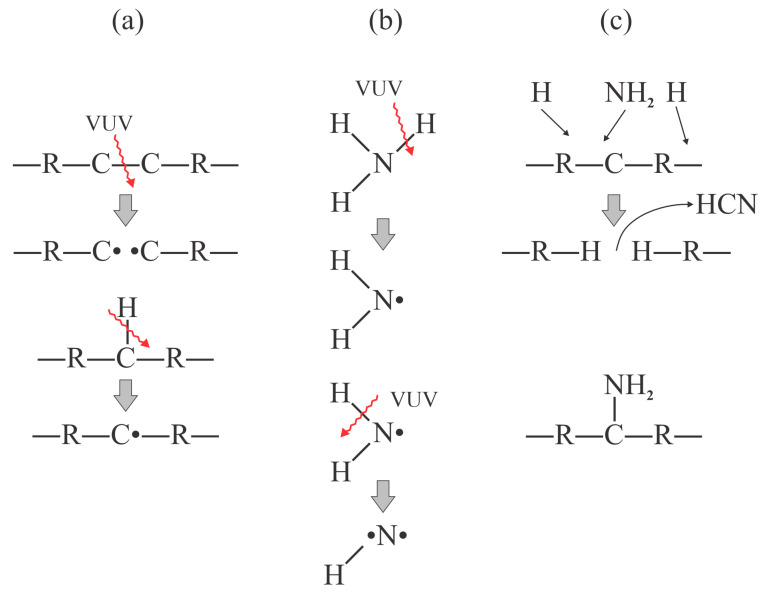
The effects of treating PP foils with VUV radiation in an ammonia atmosphere at low pressure are as follows: (**a**) VUV radiation causes the formation of dangling bonds in the surface film; (**b**) ammonia is partially dissociated by the absorption of VUV radiation; and (**c**) the net effect is functionalization with amino groups.

The team [33] compared the effects of nitrogen plasma treatment of PP foils with those obtained by irradiation with VUV photons from a Kr resonant lamp operating at 123.6 nm in an ammonia atmosphere at 40 Pa. The nitrogen plasma was sustained at atmospheric pressure by a DBD discharge operating at a frequency of a few kHz and a peak voltage of 3.7 kV. The selected energy surface density was 0.9, 1.4, 1.9, and 2.8 J/cm^2^, namely, much higher than in [32]. The surface free energy of the untreated PP foils was 27 mJ/m^2^ and increased to 37, 44, and 57 mJ/m^2^ after treating the samples at energy densities of 0.3, 1, and 2 J/cm^2^, respectively. They found that VUV radiation was beneficial, which is similar to Behnisch’s pioneering study [24]. The authors [33] reported surface energy as high as 65 mN/m, and the concentration of nitrogen, as determined by XPS, as high as 24 at.% after treating the PP samples with VUV radiation for 2800 s. Shorter treatment times led to significantly different results. Treatment with VUV radiation in an ammonia atmosphere for 500 s caused marginal surface effects. The pioneering work on VUV-stimulated surface hydrophilization showed promising results in terms of stability since the hydrophobic recovery was relatively small. The surface energy of 65 mN/m quickly dropped to 60 mN/m but remained as high as 55 mN/m even after storing the samples under ambient conditions for 3 months. Powerful VUV sources are available these days and thus a treatment time of 1 s is sufficient [34]. The effects reported in [33] are presented in Figure 5. The first dissociation energy of ammonia is 2.6 eV [35]; therefore, VUV photons with an energy of 10 eV can be absorbed and cause the formation of NH_x_ radicals. Since the NH_3_ pressure is quite low, many photons reach the PP surface, causing bond scission in the surface film. The NH_x_ radicals occupy the dangling bonds and form stable nitrogen-containing functional groups. Therefore, the modified film is more than a monolayer thick, which explains the large nitrogen concentration as determined by XPS, the large surface energy as determined by WCA, and the slow hydrophobic recovery.

**Figure 6:**
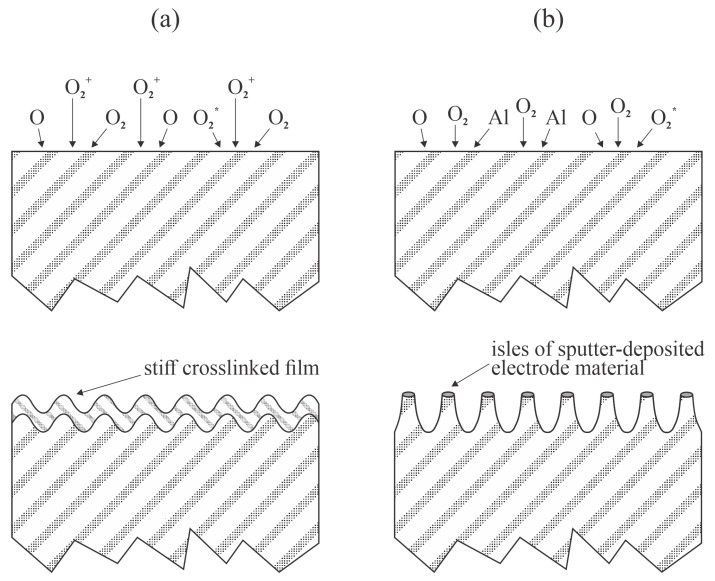
Possible mechanisms of formatting highly wettable PP after prolonged treatment with plasma sustained by low-frequency capacitively coupled RF discharge at low pressure: (**a**) buckling instability; and (**b**) the masking effect of deposited material sputtered from the biased electrode; O_2_* denotes excited oxygen molecule species.

Ren et al. [36] treated PP foils and nonwoven fabrics with low-pressure oxygen plasma sustained by a capacitively coupled RF discharge at 40 kHz. The oxygen pressure was kept constant at 15 Pa, whereas the discharge power was varied between 200 and 600 W, and the treatment time was up to 8 min. The WCA was measured for foils only given that the droplets were absorbed in the oxygen plasma-treated fabrics. At a discharge power of 600 W, the WCA decreased from 100° to approximately 30° after 1 min of plasma treatment, remained fairly unchanged for another 6 min, and dropped to 10° after treatment for 8 min. At a fixed treatment time of 4 min, the WCA was 40° at 200 W and approximately 30° for discharge powers between 300 and 600 W. XPS characterization was performed 2 days after the plasma treatment. The surface film of the plasma-treated samples contained about 30 and 25 at.% of oxygen for foils and PP fabrics, respectively. No statistically significant differences in composition were observed 22 days after the plasma treatment. Significant hydrophobic recovery was observed with both the foils and fabrics. The WCA on the foil increased to 57° and 71° after 12 days and 1 month of storage in ambient conditions, respectively. Interestingly, the water droplets were absorbed in the fabrics made from PP fibers with a rich morphology on the micrometer scale, even after having been stored for 1 month. Ren et al. [36] clearly showed that the hydrophobic recovery of PP is not correlated with the loss of oxygen on the polymer surface. The paper by Ren et al. [36] is the only one to have reported very low WCA on PP foils (10°), and explained it by increasing roughness after prolonged treatment. Unfortunately, the team did not report the discharge voltage to estimate the kinetic energy of ions impinging on the electrodes, or the position of the foils. If the foils were placed onto an electrode of a low-frequency RF discharge, a DC self-biasing would occur, and the foil would be subjected to bombardment by energetic ions, as displayed in Figure 1. Namely, the sheath is likely to be almost collisionless at a pressure of 15 Pa, where the mean free path is about 1 mm [37]. Prolonged bombardment with positive ions is likely to cause nanostructuring, as explained in detail by Bruce et al. [38]. Alternatively, bombardment of the powered electrode by positively charged ions causes sputtering. The sputtered atoms condense on the surfaces, forming unevenly distributed metal-oxide clusters that locally prevent polymer etching. As an outcome, a nanostructured surface is formed, as elaborated by Tsougeni et al. [39] and Palumbo et al. [40]. Both effects are illustrated in Figure 6.

Encinas et al. [41] treated PP foils with gaseous plasma at a pressure of 2 bar. Plasma was sustained using a rotating torch powered by a 20 kV voltage source operating at a frequency of 17 kHz (Plasma Treat GmbH, Steinhagen, Germany) and expanded to the ambient pressure in which the polymer foils were placed. Plasma-treated foils were stored in dust-free air at a relative humidity of approximately 50% and a temperature of 25 °C. The untreated PP exhibited a surface free energy of 22 mJ/m^2^ (corresponding to a WCA of approximately 100°), and the plasma treatment caused an increase to 50 mJ/m^2^ (the WCA dropped to ~42°). While this surface free energy (SFE) persisted for a few days of storage, prolonged aging (21 and 32 days) caused the SFE to increase. Such a rise in the surface energy following storage has not been reported by other authors and has yet to be explained. All other authors reported a decrease in surface energy following storage under ambient conditions. A large standard deviation was reported for PP foils aged for different periods of up to about 1 week. The WCA after 1 day of aging was approximately 45°, which decreased to 40° after 1 week. The lowest WCA of about 20° was reported for a sample stored for 1 month. This is one of the few scientific articles to report a larger SFE (lower WCA) for aged PP foils than for as-treated foils. The unexpected behavior of the surface wettability was supported by XPS measurements. The authors found that the concentration of the hydroxyl groups was approximately 12 at.%, even for an untreated sample. Plasma treatment caused an increase in the concentration to 30 at.%, and the formation of C=O as well as O–C=O functional groups among the concentration of about 10 at.%. The highest concentration of oxygen, as calculated from the survey spectra, was reported for samples stored for 21 days.

Chen et al. [42] treated PP foils with oxygen plasma sustained by a capacitively coupled RF discharge at a pressure of 60 Pa. The aging of plasma-treated samples was studied for samples stored in either in a vacuum desiccator or distilled water. The samples aged in water were dried well before the water contact angle was measured. The WCA of the untreated PP sample was 103°. The plasma treatment caused a gradual decrease in the WCA when the plasma was sustained at a discharge power of 20 W, and it was more rapid when sustained at 80 W. The final WCA after 10 min of plasma treatment was approximately 90° for all discharge powers. A discharge power and treatment time of 80 W and 120 s, respectively, were found to be optimal because the lowest WCA of 80° was obtained. However, aging was studied for samples that exhibited a WCA of 56° immediately after plasma treatment. When stored in air, these samples exhibited weak hydrophobic recovery within the first week, but prolonged storage in air did not influence the wettability as the WCA remained at approximately 70° for a period between 10 and 90 days after the plasma treatment. Somewhat different behavior in surface wettability was observed for plasma-treated PP samples stored in distilled water. The sample was immersed in water for a very short time, dried in a vacuum, and probed for wettability. Even brief immersion in water led to an increase in the WCA from 56 to 73°. Storage in water for a few days caused further hydrophobic recovery because the WCA for the samples stored in water increased to approximately 80° within 1 week. Thereafter, it remained constant for the next 80 days. The rapid hydrophobic recovery of PP samples stored in water may be explained by the removal of highly functionalized molecular fragments, as displayed in Figure 4.

The behavior of washed PP samples reported by Chen et al. [42] was observed to be similar in Jokinen et al. [43]. Jokinen used a low-pressure plasma reactor powered by a microwave (MW) discharge at a frequency of 2.45 GHz at a power of 500 W. The gas flow rate was 800 sccm, but neither the gas pressure nor the pumping speed of the vacuum pump was provided. The WCA of the untreated PP foils was 101°, which decreased to ~60° after treatment with oxygen plasma for 10 min. The samples immersed in water exhibited very fast and total hydrophobic recovery, which may be explained by the removal of the surface-modified moieties upon washing (Figure 4). Jokinen et al. [43] also treated the same PP samples with nitrogen plasma and found somewhat better wettability since the WCA after treating for 10 min dropped to 43°. Still, hydrophobic recovery was faster for these samples. All washed samples eventually assumed the WCA typical for non-treated samples, even after being stored for a few days. The samples stored in air did not exhibit total hydrophobic recovery because the WCA remained at 85–90° even after aging for 120 days.

Kalapat et al. [44] treated biaxially oriented PP foils with atmospheric pressure plasma sustained by a corona discharge in the air. The WCA was measured on samples treated at different discharge powers, normalized on the surface area between about 1.5 and 76 J/cm^2^. The WCA for the untreated samples was 100° and decreased to 72, 65, and 55° after receiving energy doses of 1.5, 3.8, and 7.6 J/cm^2^, respectively. No significant aging was observed after the samples were stored in ambient conditions for up to 3 months. The stability of the moderately hydrophilic character of these samples is difficult to explain, especially since Novak et al. [30] used a similar discharge but reported a fast hydrophobic recovery.

Ozkaya et al. [45] used an MW discharge in the electron cyclotron resonance mode to sustain the plasma, which is useful for the surface hydrophilization of PP films. The films were deposited onto gold-coated silicon wafers via spin coating. The samples were mounted in a vacuum chamber and exposed to oxygen ions extracted from the plasma using an appropriate electrically biased grid. The ion kinetic energy after impinging on the polymer samples was 250 eV, and the flux at the beam center was approximately 5 × 10^17^ m^−2^s^−1^. The evolution of the surface morphology must be as illustrated in Figure 6a. The oxygen content on the PP surface, as probed by XPS, increased with higher doses of oxygen ions. AFM-based chemical force microscopy revealed an increased adhesion force after receiving an oxygen ion fluence of approximately 5 × 10^17^ m^−2^. Nevertheless, the adhesion force was not significantly different from that of the untreated sample at other fluences, up to 3 × 10^19^ m^−2^.

Kostov et al. [46] used two different discharges for treating PP foils: an atmospheric-pressure plasma jet sustained in argon and a dielectric barrier discharge (DBD) sustained in air. The plasma jet was operated at a voltage of 10 kV, frequency of 37 kHz, and treatment time of 1 min. DBD was operated at 12 kV and 20 kHz. XPS was used to determine the changes in the surface composition. The untreated samples contained 2 at.% of oxygen, and the high-resolution C1s peak revealed that oxygen was bonded in the hydroxyl groups. Treatment with the plasma jet caused an increase in the oxygen content to 27 at.%, and approximately one-third of the oxygen was bonded to the O–C=O groups. The lowest achievable WCA was 52°. Rinsing in distilled water for 1 min caused significant changes in the surface composition because the HR XPS C1s peak corresponding to O–C=O vanished and the peak corresponding to the C=O group was close to the detection limit. The authors concluded that only molecular fragments were heavily oxidized after the plasma treatment and were removed by rinsing with distilled water. The effect is shown in Figure 4. Similar results were reported for PP treated by DBD, except that the oxygen concentration after the plasma treatment was only ~16 at.%. Interestingly, the rinsing was not as effective as for the samples treated by the plasma jet since the concentration of the remaining hydroxyl groups was almost twice that of those treated with the plasma jet. The much higher degree of functionalization while using Ar plasma can be explained by the VUV radiation arising from the plasma [47]. Radiation causes bond scission and the formation of numerous dangling bonds in the surface film. The dangling bonds interact with oxygen from the effluent gas or water vapor, which desorbs from the tube in which the jet was sustained [48].

Lindner et al. used atmospheric pressure corona discharge in air to treat PP foils [49]. The discharge energy normalized to the surface area was in the range of 1–5 J/cm^2^. The WCA of the untreated samples was approximately 100° and decreased to about 72° independent of the normalized discharge energy. Gradual hydrophobic recovery was observed upon aging under ambient conditions. However, the measured WCAs were significantly scattered, and the error bars were large. The average WCAs after aging for 1 day, week, and month were approximately 72, 75, and 77°, respectively. Half a year of aging led to a further increase in WCA to approximately 80°.

Matoušek et al. [50] treated PP foils in air plasma. The DBD discharge was powered by a generator at a frequency of 3 kHz, voltage of 20 kV, and power of 120 W. The selected treatment times were 1, 2, and 3 s. The water contact angle of the untreated sample was approximately 99°. The WCA dropped after the plasma treatment to 81, 75, and 70° for treatment times of 1, 2, and 3 s, respectively. An increase in wettability is expected for short treatment times. Of greater interest is the report on the hydrophobic recovery. The WCA for all three samples decreased 1 day after the plasma treatment and assumed values of 67, 69, and 67°, respectively. Such an increase in wettability after storing plasma-treated polymers for 1 day under ambient conditions was reported by Encinas et al. [41]. The XPS results, on the other hand, showed a gradual decrease in the oxygen concentration during aging, which contradicts what was reported by Ren et al. [36]. About 6 at.% of oxygen was found on the surface of an untreated PP foil, and 17% just after treatment. The oxygen concentration decreased to 12 at.% after 2 months of aging, irrespective of the plasma treatment duration. Aging was established to be detrimental to the adhesion of the solvent-based paint because it was within the limits of the experimental error for the untreated samples. The reported adhesion of water-based paint followed a path that is difficult to explain.

**Figure 7:**
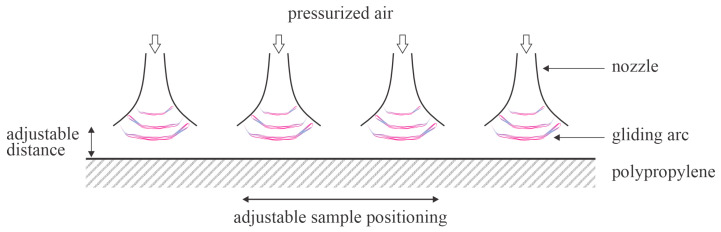
Illustration of a scalable method for almost optimal activation of two-dimensional PP samples.

Pichal et al. [51] treated thick PP foils with plasma in air at atmospheric pressure, sustained by a gliding arc in a specific configuration. Four linearly placed gliding arcs were mounted parallel to each other so the total length of the plasma in contact with the polymer was about 20 cm. The discharge power was about 1 kW, but the PP sample was placed away from the arcs; the thermal effects were thus suppressed. The samples were mounted on a conveyor belt and moved at various speeds from 0.05 to 0.2 m/s. The described configuration enabled the plasma treatment time of the PP plate to be approximately 1 s at a belt speed of 200 mm/s. The gliding arcs occurred at the exhaust of the pressurized air nozzle. Dry air was pressurized to 6.5 bar and delivered to the arcs at a flow rate of 70 L/s. As expected, the surface wettability of the PP samples decreased with increasing distance from the main discharges and with increasing conveyor belt speed. The WCA of the untreated PP sample was 102°. The minimal WCA after plasma treatment of 25° was reported for the distance between the arc discharge PP plate of 5 mm at a conveyor belt speed of 50 mm/s. This is namely the lowest value of the water contact angle reported by any team that published experimental results on the hydrophobic recovery of PP treated for reasonable times. Further, such a low WCA persisted for a few days in the air at 23 °C and 50% humidity. The error bars were moderate at approximately ±10°. Hydrophobic recovery became significant only after storing the plasma-treated sample for 10 days, whereas 110 days of storage resulted in serious, yet incomplete recovery since the WCA was approximately 85°. Samples treated at a higher conveyor belt speed did not perform well because the WCA after plasma treatment was close to 50°, which is a value typically reported by most authors. The methods disclosed by Pichal et al. [51] thus enable almost optimal wettability of PP samples and are scalable virtually to any dimensions. One drawback of this method is its high energy consumption. The method is illustrated in Figure 7.

**Figure 8:**
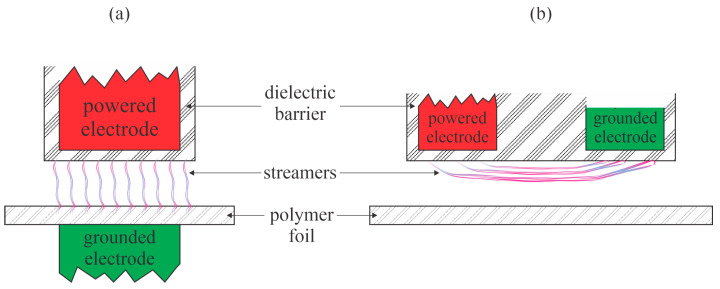
Illustration of: (**a**) standard; and (**b**) coplanar DBD for the treatment of two-dimensional PP samples.

Šramkova et al. [20] performed hydrophilization of PP foils with atmospheric pressure plasma sustained in the air by a couple of discharges: a conventional DBD and a coplanar DBD. The WCA of the untreated samples was 105°. The main difference between these discharges is that the discharge streamers hit the surface of the polymer samples upon plasma treatment while using standard DBD, and did not hit the polymer surface in the case of coplanar discharges. Thus, the classical DBD always causes thermal damage, including microcraters at positions where they hit the polymer surface, whereas the coplanar discharges enable gentler surface treatment because the major reactants are molecular radicals that move randomly. The treatment time was 1–10 s. The conventional DBD caused a minimal WCA of approximately 68° at a treatment time of 3 s. The coplanar DBD, however, caused a monotonous decrease in the WCA, which was 87, 63, 60, and 53° after treating the PP foils for 1, 3, 5, and 10 s, respectively. Modest hydrophobic recovery was reported after storing the plasma-treated samples under ambient conditions for up to 1 month. A comparison of the standard and coplanar DBD is provided in Figure 8. Configuration (Figure 8b) prevents polymer damage by streamers, but the distance between the electrodes and the polymer foil should be adjusted carefully due to the large vertical gradients in the concentration of the reactive gaseous species.

Table 1 presents a summary of the key results reported in the reviewed articles. All authors reported the type of discharge selected for sustaining gaseous plasma, as well as the gas or gas mixture. While many authors also reported the discharge power, it should be stressed that the samples were usually much smaller than the area of the plasma-surface interface and thus discharge power alone might not be the relevant parameter. The authors also reported the treatment time, and the WCA before and after plasma treatment. A few authors also studied the hydrophobic recovery of plasma-treated polypropylene samples.

## 5. Wettability Versus Plasma Treatment Time

**Figure 9:**
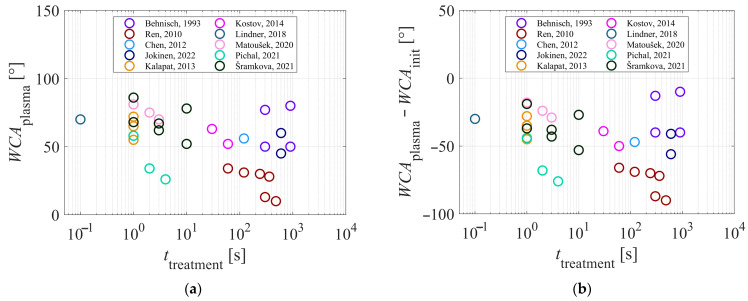
(**a**) WCA after plasma treatment versus plasma treatment time; and (**b**) difference in WCA between the untreated and plasma-treated samples. Source: [20,24,36,42,43,44,46,49,50,51].

The results summarized in Table 1 provide the basis for drawing any correlation between the processing parameters and the resultant wettability as well as hydrophobic recovery. Almost all authors reported the plasma treatment time. The WCA measured soon after plasma treatment is shown in Figure 9a. The WCA for the untreated samples varied by more than 10°, which might have screened the plasma effect. Therefore, Figure 9b shows the difference between the WCA of the untreated and plasma-treated samples versus the treatment time.

The results reported by various authors are scattered, which could be explained by the various treatment configurations. It is interesting that there is practically no correlation between the wettability and the treatment time. That is, the average WCA after the plasma treatment (Figure 8a) is approximately 60°, and this WCA could be achieved in either 100 ms of plasma treatment, as disclosed by Lindner et al. [49], or several minutes, as disclosed by Behnisch et al. [24]. These two sets of authors used completely different discharges to sustain gaseous plasma. Lindner et al. [49] treated PP foils passing a powerful (power per volume) discharge at a speed of about 0.1 m/s, whereas Behnisch et al. [24] treated samples away from glowing plasma.

Even though the increase in surface wettability should depend on the dose of plasma species, none of the authors reviewed in Table 1 reported it. Some authors used different treatment times in the same reactor, and the wettability often rose with increased treatment time, as disclosed, for example, by Matoušek et al. [50] and Ren et al. [36]. Such behavior, however, cannot be viewed to be a general rule because Figure 8b and Figure 9a show no correlation between the WCA and treatment time. A possible explanation for this paradox may be that most authors selected treatment times that were too long and in so doing missed the initial stages of surface hydrophilization. In a pioneering study, Dorai et al. [14] reported that the PP surface was saturated with polar groups after treating the sample with a powerful corona discharge, already after a microsecond. More recently, Vesel et al. [52] systematically treated selected aromatic polymers with the afterglow of oxygen plasma and reported optimal wettability after 1 s, provided that the O-atom density was large. In fact, they found a minimal WCA after receiving a fluence of O atoms of 10^23^ m^−2^. The treatment times in glowing oxygen plasma should be much lower, and so should the fluences, due to the synergistic effects with other plasma species, as illustrated in Figure 1. The evolution of the surface hydrophilicity of PP versus the fluence of O atoms has yet to be reported, as have the synergies with ions and VUV radiation. The latter should boost the evolution of wettability, as reported by Behnisch et al. [24] and Truica-Marescu et al. [29,33].

## 6. Effect of Washing

A few teams exposed plasma-treated polyethylene to a liquid, either ethanol or water. Some rinsed the polymer samples, while others simply immersed them in liquid. All authors reported an immediate loss in surface wettability due to the water exposure. The effects are presented in Table 2.

Table 2 shows a variety of water contact angles measured for untreated PP samples. The WCA spans across a broad range, from 85 to 103°. This may be due to the different synthesis procedures of PP foils, while another feasible explanation considers surface impurities. The uncertainty in measuring the WCA can be excluded because such large differences are visible to the naked eye. In addition, the WCA after plasma treatment varied significantly, from 42 to 65°. Such large differences could be explained by the various discharges used by different authors, which produce plasmas with different parameters, as already discussed in this article. Of greater interest is the finding that the WCA of the plasma-treated and washed samples did not vary significantly, only from 73 to 81°. This observation is strange at a glance. Still, a feasible explanation of the almost independent wettability after the washing of plasma-treated polyethylene samples is as follows: plasma treatment for 1 min or longer causes etching and thus the removal of the surface film, whose thickness varies with the discharge parameters. The surface of the plasma-treated PP is covered with molecular fragments after the prolonged plasma treatment, as illustrated in Figure 4b. The discharge conditions should influence the composition and structure of the fragments, but the modification of the underlying PP should be fairly independent of the discharge conditions as it receives a rather small dose of reactive plasma species, even though the flux onto the surface (i.e., molecular fragments) varies enormously depending on the type of discharge used to sustain gaseous plasma. According to this hypothesis, the wettability of PP after washing should be similar to that of pure PP treated with a gentle plasma for a short time. Even doses of radicals of the order of 10^20^ or less will cause significant functionalization with polar groups, as shown for another polyolefin in a classical paper by Kushner et al. [53].

## 7. Hydrophobic Recovery

As mentioned above, the high wettability of polymer materials is never permanent; the loss of hydrophilicity occurs spontaneously even after being stored in vacuum. Given that such storage is impractical, most authors studied hydrophobic recovery under ambient conditions, that is, room temperature and moderate humidity. Different teams reported different aging times, with Table 1 showing the WCA reported after 1 day, 1 week, and 1 month. While these time spans may be interesting for practical applications, that is not so much the case with scientific research since the WCA increase follows logarithmic behavior for many polymers [9,54]. The super-hydrophilic surface finish of many polymers is actually lost within minutes or hours after the plasma treatment [55].

**Figure 10:**
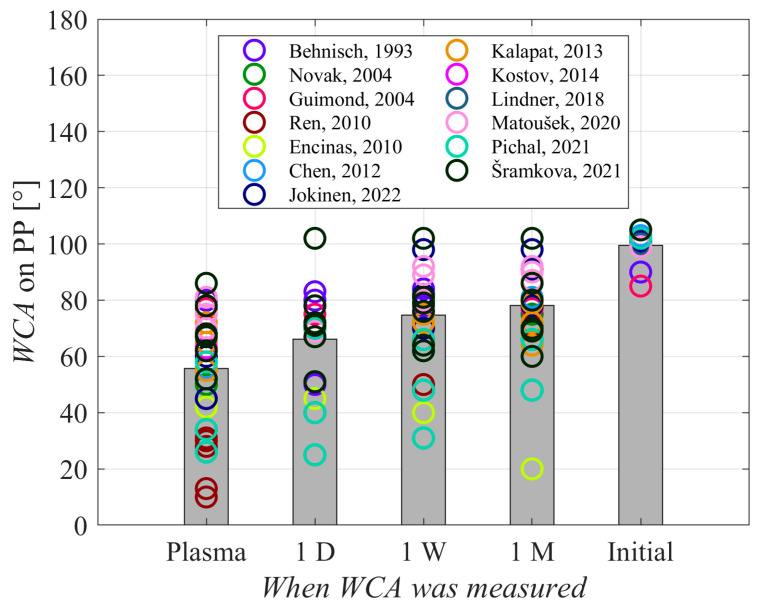
Hydrophobic recovery of plasma-treated PP; “Initial” indicates WCA before plasma treatment, “Plasma” indicates WCA just after plasma treatment, and “1 D”, “1 W”, and “1 M” indicate WCAs of plasma-treated samples aged for 1 day, 1 week, and 1 month, respectively. Gray bars indicate the average WCA values of displayed aspects. Source: [20,24,30,32,36,41,42,43,44,46,49,50,51].

Figure 10 presents a plot of water contact angles measured just after the plasma treatment (1st column marked “plasma”), 1 day after plasma treatment, 1 week, and 1 month after the plasma treatment (2nd, 3rd, and 4th columns, marked “1 D”, “1 W”, and “1 M”, respectively). The right-most column (5th column marked “Initial”) represents the water contact angles measured for untreated samples. The samples were not washed after plasma treatment. The height of the column is the average value, and the values reported by different authors are represented by circles. The values reported by Encinas et al. [41] were excluded from the average as it is impossible to explain the large deviation from the values reported by other authors.

As expected, hydrophobic recovery causes a gradual increase in the water droplet contact angle and thus a decrease in the surface wettability. The results reported by various authors are scattered, but the general trend is clear. The WCA after aging for 1 month is still lower than that of the untreated samples. This is explained by the persistence of some polar functional groups on the polymer surface, even after prolonged aging. The majority of the functional groups might have re-oriented towards the bulk, and oxygen may have diffused in the subsurface film, yet the aging still does not cause complete hydrophobic recovery in the time span up to 1 month. The hydrophobic recovery is accelerated upon heating the polymer samples. For example, Oh et al. [56] stored plasma-treated polyethylene terephthalate samples at different temperatures and reported complete hydrophobic recovery within 1 h after plasma treatment if the samples were stored in the air at a temperature of 400 K or higher. In fact, the samples stored at such high temperatures assumed a WCA much larger than before any treatment, which was explained by nanostructuring caused by plasma treatment. Alternatively, storage at temperatures well below room temperature is likely to delay hydrophobic recovery, but no authors have ever reported such experiments for polypropylene. In any case, storage at low temperatures is impractical.

## 8. Conclusions

Relevant literature on the stability of plasma-hydrophilized PP was reviewed. Although some articles showed the PP wettability varied with the processing parameters, a comparison of results reported in different articles indicates no conclusive correlation with the reported processing parameters, such as treatment time, type of discharge used for sustaining plasma, the discharge power, type of gas, gas pressure, or flow, etc. The reported water contact angles for untreated PP samples span from 85 to 105°, which may be due to the synthesis procedure and inclusion of other materials, or simply to surface impurities. The WCA after plasma treatment varies by a factor of 3 or more, which is explained by the formation of surface molecular fragments whose composition and structure vary significantly with the dose of reactive plasma species.

The results presented by all authors show clearly that the surface wettability of washed plasma-treated samples is practically independent of the processing parameters, which may be an important direction for future scientific and technological work on the plasma activation of PP. The molecular fragments are routinely removed by rinsing or even storing plasma-treated PP in water. This peculiarity of PP dictates short treatment times to suppress the formation of loosely bonded molecular fragments, thereby facilitating the adhesion of various coatings to the PP surface. The directions have yet to be disclosed in the scientific literature notwithstanding the fact that the industry has been routinely using the plasma technique to modify PP foils for decades.

## Figures and Tables

**Table 1:** Summary of reported processing parameters and evolution of the water contact angle upon storage of plasma-treated polypropylene.

Author	PP Type	Discharge	Gas	*p* [Pa]	*P* [W]	*t*_treat_ [s]	WCA_I_ [°]	WCA_P_ [°]	1 Day	1 Week	1 Month	Washing After Plasma	Ref.
Behnisch	Foil	CCP-RF ^a^	O_2_	50	N/A	300	90	50	50			No	[24]
Behnisch	Foil	CCP-RF ^a^	H_2_ + O_2_	50	N/A	600 + 300	90	50	50			No	[24]
Behnisch	Foil	CCP-RF ^a^	O_2_	50	N/A	300	90	77	80	78	80	Ethanol ultrasound	[24]
Behnisch	Foil	CCP-RF ^a^	H_2_ + O_2_	50	N/A	600 + 300	90	80	83	84	80	Ethanol ultrasound	[24]
Novak	B-foil ^b^	Corona	Air	10^5^	N/A ^i^	N/A	N/A	65			70	No	[30]
Novak	E-foil ^c^	Corona	Air	10^5^	N/A	N/A	N/A	50			75	No	[30]
Guimond	Foil	Corona	Air	10^5^	N/A	N/A	85	65	75		77	No	[32]
Guimond	Foil	Corona	Air	10^5^	N/A	N/A	85	77				Deionized water	[32]
Ren	Foil	CCP-RF	O_2_	15	600	480	100	10				No	[36]
Ren	Foil	CCP-RF	O_2_	15	600	360	100	28				No	[36]
Ren	Foil	CCP-RF	O_2_	15	600	240	100	30				No	[36]
Ren	Foil	CCP-RF	O_2_	15	600	120	100	31				No	[36]
Ren	Foil	CCP-RF	O_2_	15	600	60	100	34				No	[36]
Ren	Foil	CCP-RF	O_2_	50	600	300	100	13		50	71	No	[36]
Encinas	Foil	Jet ^d^	Air	2 × 10^5^	N/A	N/A	100	42	45	40	20	No	[41]
Chen	Foil	CCP-RF	O_2_	60	80	120	103	56		70	70	Stored in air	[42]
Chen	Foil	CCP-RF	O_2_	60	80	120	103	56		80	81	Stored in water	[42]
Jokinen	Foil	MW ^e^	O_2_	N/A	500	600	101	60		70	78	Stored in air	[43]
Jokinen	Foil	MW	O_2_	N/A	500	600	101	60		98	98	Stored water	[43]
Jokinen	Foil	MW	N_2_	N/A	500	600	101	45		82	91	Stored in air	[43]
Jokinen	Foil	MW	N_2_	N/A	500	600	101	45		102	102	Stored in water	[43]
Kalapat	B-foil	Corona	Air	10^5^	N/A	1	100	72		75	79	Stored in air	[44]
Kalapat	B-foil	Corona	Air	10^5^	N/A	1	100	65		72	72	Stored in air	[44]
Kalapat	B-foil	Corona	Air	10^5^	N/A	1	100	55		62	64	Stored in air	[44]
Kostov	Foil	Jet	Ar	10^5^	N/A	60	102	52				Stored in air	[46]
Kostov	Foil	DBD ^f^	Air	10^5^	N/A	30	102	63				Stored in air	[46]
Kostov	Foil	Jet	Ar	10^5^	N/A	60	102	52				Stored in water	[46]
Kostov	Foil	DBD	Air	10^5^	N/A	30	102	63				Stored in water	[46]
Lindner	B-foil	Corona	Air	10^5^	800	0.1	100	70	72				[49]
Matoušek	Foil	DBD	Air	10^5^	N/A	1	99	81	67	89	92		[50]
Matoušek	Foil	DBD	Air	10^5^	N/A	2	99	75	69	92	90		[50]
Matoušek	Foil	DBD	Air	10^5^	N/A	3	99	70	67	80	87		[50]
Pichal	Plate	G-arc ^g^	Air	10^5^	1000	4	102	26	25	31	48		[51]
Pichal	Plate	G-arc	Air	10^5^	1000	2	102	34	40	48	66		[51]
Pichal	Plate	G-arc	Air	10^5^	1000	1	102	58	70	66	70		[51]
Šramkova	B-foil	CP-DBD ^h^	Air	10^5^	400	1	105	86	102	102	102		[20]
Šramkova	B-foil	CP-DBD	Air	10^5^	400	3	105	62	72	79	70		[20]
Šramkova	B-foil	CP-DBD	Air	10^5^	400	10	105	52	51	62	60		[20]
Šramkova	B-foil	DBD	Air	10^5^	380	1	105	68	67	64	69		[20]
Šramkova	B-foil	DBD	Air	10^5^	380	3	105	67	71	76	80		[20]
Šramkova	B-foil	DBD	Air	10^5^	380	10	105	78	78	81	86		[20]

^a^ Capacitively coupled radiofrequency plasma; ^b^ biaxially oriented foil; ^c^ extruded foil; ^d^ atmospheric-pressure plasma jet; ^e^ microwave plasma; ^f^ dielectric barrier discharge; ^g^ gliding arc plasma; ^h^ coplanar dielectric barrier discharge; ^i^ not available.

**Table 2:** Reported water contact angles for the PP samples before treatment, immediately after plasma treatment, and immediately after washing the plasma-treated samples.

Conditions	Initial WCA [°]	WCA After Plasma [°]	WCA After Washing [°]	Author
Treatment for about 5 min in O_2_ plasma afterglow at 50 Pa; dipping into 96% ethanol in an ultrasound cleaner for 5 min	90	50	77	Behnisch [24]
Pre-treatment with H_2_ plasma afterglow at 50 Pa for 10 min, followed by treatment for about 5 min in O_2_ plasma afterglow at 50 Pa; dipping into 96% ethanol in an ultrasound cleaner for 5 min	90	50	81	Behnisch [24]
Treatment with corona in air at 1 bar; immersed in water for 1 min	85	65	77	Guimond [32]
Treatment with DBD sustained in N_2_ at 1 bar; immersed in water for 1 min	85	42	74	Guimond [32]
Treatment for 2 min in CCP RF O_2_ plasma at 60 Pa; immersed in water for a short time (details not reported)	103	56	73	Chen [42]
Treatment for 1 min in low-pressure (exact pressure not disclosed) O_2_ plasma at 500 W, sustained by a microwave discharge; washing method not disclosed	101	60	75	Jokinen [43]

## Data Availability

No new data were generated; all data were taken from the literature.

## Abbreviations

The following abbreviations are used in this manuscript:

PP	Polypropylene
VUV	Vacuum ultraviolet
WCA	Water contact angle
XPS	X-ray photoelectron spectroscopy
SIMS	Secondary ion mass spectrometry
FTIR	Fourier-transform infrared
AFM	Atomic force microscopy
SEM	Scanning electron microscopy
RF	Radiofrequency
PCSFE	Polar component of the surface free energy
SFE	Surface free energy
MW	Microwave
DBD	Dielectric barrier discharge
